# The Structure–Antioxidant Activity Relationship of Ferulates

**DOI:** 10.3390/molecules22040527

**Published:** 2017-03-25

**Authors:** Magdalena Karamać, Lidiya Koleva, Vessela D. Kancheva, Ryszard Amarowicz

**Affiliations:** 1 Department of Chemical and Physical Properties of Food, Institute of Animal Reproduction and Food Research, Polish Academy of Sciences, Tuwima 10, 10-748 Olsztyn, Poland; m.karamac@pan.olsztyn.pl; 2 Lipid Chemistry Department, Institute of Organic Chemistry with Centre of Phytochemistry, Bulgarian Academy of Sciences, Sofia 1113, Bulgaria; lidiya.koleva@gmail.com (L.K.); vessy.kancheva@abv.bg (V.D.K.)

**Keywords:** ferulates, structure, antioxidant activity, 2,2′-azinobis-(3-ethylbenzothiazoline-6-sulfonic acid) (ABTS), ferric-reducing antioxidant power (FRAP), lipid oxidation

## Abstract

The antioxidant activity of ferulic acid (**1**), *iso-*ferulic acid (**2**), coniferyl aldehyde (**3**), methyl ferulate (**4**), and ethyl ferulate (**5**) were investigated using 2,2′-azinobis-(3-ethylbenzothiazoline-6-sulfonic acid) (ABTS) and ferric-reducing antioxidant power (FRAP) assays and autoxidation of triacylglycerols of commercially available sunflower oil (TGSO). The compounds tested for ability to scavenge ABTS radical cations was in the order of ferulic acid > coniferyl aldehyde ≈ *iso-*ferulic acid > ethyl ferulate ≈ methyl ferulate. The results of the FRAP assay for ferulic acid, *iso-*ferulic acid, and coniferyl aldehyde were similar to and higher than those of methyl ferulate and ethyl ferulate. In the lipid system, *iso-*ferulic acid showed weak antioxidant activity. The other ferulates exhibited much stronger, yet similar, activities.

## 1. Introduction

Phenolic acids and their derivatives have potential biological function. They are widely spread throughout the plant kingdom and in foods of plant origin. The antioxidant properties of phenolic acids depends on their chemical structure. Cinnamic acid derivatives have been characterized as more potent than benzoic acid derivatives, due to the resonance stabilization enhanced by the conjugation between π electrons of the ring and the π bond of the side-chain [1]. The *ortho*-dixydroxyl substitution pattern is commonly regarded as important for the radical scavenging activities of phenolic acids [2,3]. The antiradical activity of phenolic acids is positively associated with methoxy groups no matter the substituent position [3].

Ferulic acid and its derivatives have been widely studied for their beneficial health properties against inflammations, diabetes, cancer, cardiovascular disease, and Alzheimer’s disease [4,5,6]. Ferulates are natural phenolic phytochemicals that are present in the peels of fruits and vegetables, the bran of grains, seeds, and leaves [4,7,8,9,10,11,12,13,14]. They can be found in plants in free form and covalently bound to plant biological polymers in plant cells. In rice bran oil, ferulic acid is esterified with sterols as γ-oryzanol [4].

Ferulates exhibit antioxidant activity in response to free radicals. One hydrogen atom from their hydroxyl group can react with a free radical. The antioxidant properties of ferulic acid and ferulates were investigated using several methods, such as 2,2’-diphenyl-1-picrylhydrazyl (DPPH), 2,2′-azinobis-(3-ethylbenzothiazoline-6-sulfonic acid) (ABTS), ferric-reducing antioxidant power (FRAP), oxygen radical absorbance capacity (ORAC), and lipid oxidation in emulsions and bulk oil systems [6,15,16,17,18,19]. Urbaniak et al. [20] analyzed hydrogen atom transfer, sequential proton loss electron transfer, single electron transfer-proton transfer, and transition metal chelation by calculating antioxidant descriptors. The results indicated differences between individual ferulates in term of their structure [16,17]. In the research of Kanski et al. [21], ferulic acid exhibited much stronger antiradical properties against DPPH radical than that of vanilic acid and coumaric acid.

Free radicals play a major role in the ecology of cancer. Ferulic acid and its derivatives due to their ability to scavenge ROS and stimulate cytoprotective enzymes, can act as anticancer agents. Ferulic acid enhances the activity of UDP-glucuronosyl transferases in the liver, and the detoxification of carcinogenic compounds accelerated [22]. Ferulic acid also inhibits carcinogenesis of colon and breast cancer [23,24].

**Figure 1:**
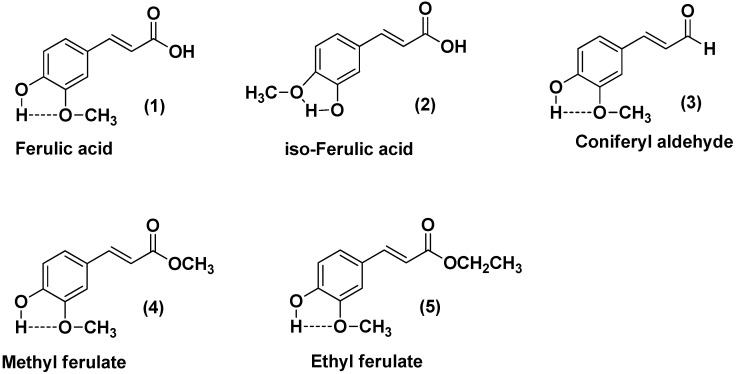
Chemical structures of the studied ferulates.

The aim of this work was to compare the antioxidant activity of ferulic acid and its four derivatives (i.e., *iso-*ferulic acid, coniferyl aldehyde, methyl ferulate, and ethyl ferulate) in polar and in lipid systems. The chemical structures of the five ferulates are depicted in Figure 1.

## 2. Results and Discussion

The antioxidant activities of ferulates were assayed using ABTS and FRAP methods. The ability of the tested compounds to scavenge ABTS radical cations was in the order of ferulic acid > coniferyl aldehyde ≈ *iso-*ferulic acid > ethyl ferulate ≈ methyl ferulate (Table 1). The effect depended on the carbon side chain groups and the position of the hydroxyl and methoxyl groups in the ring. Ferulic acid was a much stronger antioxidant than methyl and ethyl ferulates; ferulic acid, with an –OH moiety in the para-position, was much stronger than *iso-*ferulic, with an –OH group in the meta- position of the ring. In our previous studies, ferulic acid exhibited the strongest antiradical activity. *iso-*Ferulic acid at 10–100 nmol/assay did not scavenge DPPH radicals. Methyl ferulate and coniferyl aldehyde demonstrated similar antiradical properties [16]. 

Ferulic acid, *iso-*ferulic acid, and coniferyl aldehyde were characterized by similar FRAP results. These results were higher than those obtained for methyl ferulate and ethyl ferulate (Table 1). The results suggest that the presence of carboxyl and aldehyde groups decreases the ability of ferulate to reduce Fe^3+^.

Stronger activity of ferulic acid than that of *iso-*ferulic acid is related to the presence of a hydrophobic OCH_3_ group in the meta-position for para–OH phenolic acid [25]. In the study of Csepregi et al. [26], the absence of the 3-hydroxyl structure had a negative effect on FRAP or DPPH assays. The slightly weaker antiradical activity of ferulic acid esters in comparison to ferulic acid was observed by Anselmi et al. [27] using a DPPH assay.

**Figure 2:**
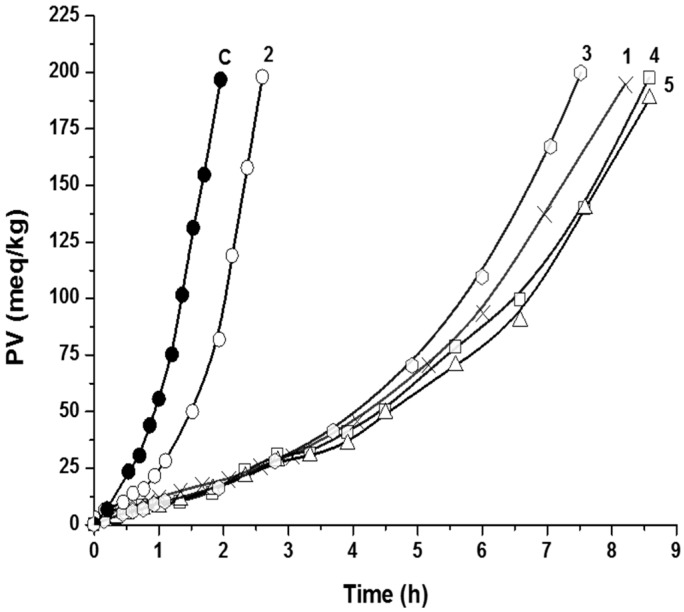
Kinetic curves of lipid hydroperoxides accumulation during triacylglycerols of sunflower oil (TGSO) autoxidation at 80 °C in the absence and in the presence of 1.0 mM of the studied compounds; C—control, 1—ferulic acid, 2—*iso-*ferulic acid, 3—coniferyl aldehyde, 4—methyl ferulate, 5—ethyl ferulate.

The results of the antioxidant activity of ferulates in the triacylglycerols of sunflower oil (TGSO) system are reported in Figure 2 and Table 2. *iso-*Ferulic acid showed weak antioxidant activity in this system. Its protection factor (PI) was only 1.3. The kinetic of TGSO autoxidation with the addition of *iso-*ferulic acid was similar to that of the control sample (Figure 2). Ferulic acid, coniferyl aldehyde, and alkyl ferulates exhibited much stronger, yet similar, activities. This was confirmed by statistical analysis. For these four phenolic compounds, the differences between the induction period in the presence of antioxidants and initial rates of lipid autoxidation in the presence of antioxidants were not statistically significant (Table 2). The good antioxidant activity of ferulic acid against TGSO has been previously reported by our group [28]. In cited work, ferulic acid at a concentration of 0.04% exhibited a protection factor (PF) of 4.2. Ferulic acid, coniferyl aldehyde, methyl ferulate, and ethyl ferulate have the same main structural fragment, which is responsible for their antioxidant potential, and they differ only in the substituent R on the side chain. However, the substituents R are far from the active center of these compounds and do not modify the antioxidant efficiency of Compounds 1 and 3–5, i.e., to scavenge LOO radicals.

In a bulk oil system at 45 °C, the antioxidant properties of ferulic acid, coniferyl aldehyde, and ethyl ferulate were similar [17]. In our previous research, in a ß-carotene linoleate model (emulsion system), the antioxidant properties of methyl ferulate were stronger than that of coniferyl aldehyde and ferulic acid. In an emulsion system, *iso-*ferulic acid exhibited the lowest activity [16]. In the study of Farhoosh et al. [29], methyl gallate and methyl *p-*hydrobenzoate showed stronger antioxidant properties in a bulk fish oil system and fish-oil-in-water emulsion. 

All compounds under study are *ortho*-methoxyphenols, with structures that are stabilized by intramolecular H-bonds. Therefore, the abstraction of H atom from their OH groups in reaction with LOO radicals is complicated, and their antioxidant potential is moderate. However, *iso-*ferulic acid, due to its meta-position of the side chain towards the OH group, has no antioxidant potential. Several investigations [30,31] have shown that ortho substitution with the methoxy group increases the stability of the aryloxyl radical and thus its antioxidant potential. Ferulic acid is a more effective antioxidant in lipohilic systems than *p*-coumaric acid because the electron-donation methoxy group allows increased stabilization of the resulting aryloxyl radical through electron delocalization after hydrogen donation by the hydroxyl group [30]. According to Koroleva et al. [32], the side chain of ferulic acid participates in the delocalization of unpaired electrons during the formation of a radical with a deprotonated carboxyl group. However, the introduction of substituents with a negative inductive effect in the benzene ring creates a steric barrier for interaction with radicals and for varies the electron density in the benzene ring. Denisov and Denisova [33] reported that oxygen-containing compounds are able to form different kinds of complexes with hydroperoxides, but only one enables accelerated hydroperoxide decomposition. Ferulic acid participates in a side reaction, increasing lipid hydroperoxide decomposition through additional chain branching reactions, in which the –COOH group is involved [33,34]: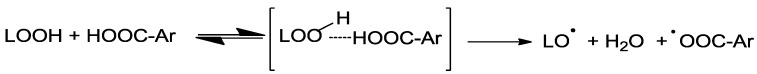.

In contrast, coniferyl aldehyde (**3**), methyl-(**4**), and ethyl-(**5**) ferulate are unable to form this type of complex (intermediates), and their molecules are not involved in additional chain branching reactions. Coniferyl aldehyde forms another type of complex, which according to Denisov and Daniseva [33] did not lead to the acceleration of lipid hydroperoxides, because the decomposition of this complex does not lead to cleavage of the O–O bond:

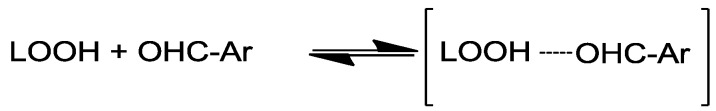
.

## 3. Materials and Methods

### 3.1. Chemicals

Methanol was acquired from P.O.Ch. Company (Gliwice, Poland). Ferrous chloride, sodium persulfate), ferulic acid, *iso-*ferulic acid, coniferyl aldehyde, methyl ferulate, ethyl ferulate, 2,2′-azinobis-(3-ethylbenzothiazoline-6-sulfonic acid) (ABTS), 2,4,6-tri(2-pyridyl)-*s*-triazine (TPTZ), and 6-hydroxy-2,5,7,8-tetramethyl-chroman-2-carboxylic acid (Trolox) were obtained from Sigma-Aldrich (Saint Louis, MO, USA). 

### 3.2. Trolox Equivalent Antioxidant Capacity (TEAC)

The TEAC was determined using the method of Re et al. [35]. ABTS**^•+^** (2,2-azino-bis-3-ethylbenzothiazoline-6-sulfonic acid cation radical) solution was prepared by mixing an ABTS in water stock solution with 2.45 mM sodium persulfate. This mixture was left for 12–16 h at room temperature in the dark until a stable oxidative state was reached. For analysis, the ABTS^•+^ stock solution was diluted with methanol to an absorbance of 0.720 at 734 nm. For the spectrophotometric assay, 2 mL of the ABTS**^•+^** solution and 20 µL of the sample solution were mixed, and the absorbance was determined at 734 nm at 37 °C for 10 min. The calibration curve was plotted using a Trolox standard. The results were expressed as mol Trolox equivalent per mol (TEAC).

### 3.3. Ferric-Reducing Antioxidant Power (FRAP)

The FRAP assay was performed as previously described by Benzie and Strain [36]. The sample solution was first diluted with deionized water to within the linearity range. The working FRAP reagent was prepared by mixing 10 volumes of a 300 mM acetate buffer, pH 3.6, with 1 volume of 10 mM TPTZ in 40 mM HCl and with 1 volume of 20 mM FeCl_3_ × 6H_2_O. A 2.25 mL volume of FRAP reagent was warmed to 37 °C. Then, 75 μL of the sample and 225 μL of deionized water were added to the FRAP reagent, and the absorbance was measured at 593 nm against a reagent blank after 30 min of incubation. The FRAP value was calculated and expressed as mmol Fe^2+^ equivalent per mol using the calibration curve of Fe^2+^.

### 3.4. Preparation of Triacylglycerols

Triacylglycerols of commercially available sunflower oil (TGSO) were cleaned of pro- and antioxidants by adsorption chromatography according to Kancheva et al. [37] and were stored under nitrogen at −20 °C. The fatty acid composition of the lipid substrate was determined by GC analysis of the methyl esters of the total fatty acids obtained using a GC-FID Hewlett-Packard 5890 instrument (Hewlett-Packard Company, Palo Alto, CA, USA) and an HP INNOWAX capillary column (polyethylene glycol mobile phase, Agilent Technologies, Santa Clara, CA, USA; 30 m × 0.25 mm × 0.25 mm). The temperature gradient started at 165 °C, increased to 230 °C at 4 °C/min, and was held constant at 230 °C for 15 min; the injection volume was 1 µL. The injector and detector temperatures were 260 and 280 °C, respectively. Nitrogen was used as the carrier gas at a flow rate of 0.8 mL/min. The analyses were performed in triplicate. Six fatty acids were present in TGSO: 16:0—6.7%; 18:0—3.6%; 18:1—25.1%; 18:2—63.7%; 20:0—0.2%; and 22:0—0.7%. Lipid samples containing various inhibitors were prepared immediately before use. Aliquots of the antioxidant solutions in purified acetone were added to the lipid sample. Solvents were removed under a nitrogen flow.

### 3.5. Lipid Autoxidation

Lipid autoxidation was performed in a thermostatic bath at 80 °C by blowing air through the samples in special vessels. The oxidation process was monitored by withdrawing samples at measured time intervals and subjecting them to iodometric determination of the primary products (lipid hydroxyperoxides, LOOH) concentration, i.e., the peroxide value (PV) [37]. All kinetic data are expressed as the average of two independent measurements, which were processed using the computer programs Origin 6.1 (OriginLab Corporation, Northampton, MA, USA) and Microsoft Excel-97 (Microsoft, Redmond, WA, USA).

### 3.6. Kinetic Parameters of the Studied Extracts and Pure Compounds

Antioxidant efficiency, induction period, protection factor (PF), inhibition degree (ID), and antioxidant capacity were calculated for the ferulates [38]. Antioxidant efficiency is the potency of an antioxidant to increase the oxidation stability of the lipid sample by blocking the radical chain process. It can be presented by the induction period*.* The PF represents how many times the antioxidant increases the oxidation stability of the lipid sample and was determined as the ratio between the induction periods in the presence (IP_A_) and absence (IP_C_) of an antioxidant, i.e., PF = IP_A_/IP_C_. ID is a measure of the antioxidant reactivity, i.e., how many times the antioxidant shortens the oxidation chain length (ID = R_C_/R_A_). Therefore, ID is one of the most important kinetic parameters. The initial rates of lipid autoxidation in the absence (R_C_) and presence of antioxidant (R_A_) were calculated on the tangent at the initial phase of the kinetic curves of hydroperoxide accumulation. Antioxidant capacity was reported using two kinetic parameters: the main rate of antioxidant consumption (R_m_) and the relative main rate of antioxidant consumption (RR_m_). R_m_ is the main rate of inhibitor consumption during the induction period of the inhibited lipid autoxidation, i.e., R_m_ = [AOH]/IP_A_. RR_m_ represents the ratio of R_m_ to R_A_, i.e., RR_m_ = R_m_/R_A_.

### 3.7. Statistical Analysis

All experiments and chemical determinations in this study were performed in triplicate. Results are reported as mean and SD values. Analyses of variance and Duncan’s test were performed at the level of *p* < 0.05 to evaluate the significance of differences among mean values.

## 4. Conclusions

1. Ferulic acid exhibited the strongest antioxidant activity investigated using ABTS and FRAP assays.
2. Methyl and ethyl ferulates showed lower results of ABTA and FRAP assays than those of ferulic acid, *iso-*ferulic acid, and coniferyl aldehyde.
3. This is the first work to compare five ferulates in terms of their antioxidant activity in the lipid system (TGSO autoxidation). *iso-*Ferulic acid showed weak antioxidant activity in this system. The other ferulates exhibited much stronger, yet similar, activities.

## Figures and Tables

**Table 1:** Antioxidant activity of ferulic and *iso-*ferulic acids, coniferyl aldehyde, and alkyl ferulates determined using 2,2′-azinobis-(3-ethylbenzothiazoline-6-sulfonic acid) (ABTS) and ferric-reducing antioxidant power (FRAP) methods.

Ferulates	TEAC (mol Trolox/mol)	FRAP (mol Fe^2+^/mol)
Ferulic acid	1.948 ± 0.056 ^a^	4.727 ± 0.139 ^a^
*iso-*Ferulic acid	1.063 ± 0.089 ^b^	4. 378 ± 0.123 ^b^
Coniferyl aldehyde	1.087 ± 0.063 ^b^	4.606 ± 0.080 ^a^
Methyl ferulate	0.904 ± 0.070 ^c^	3.469 ± 0.117 ^c^
Ethyl ferulaty	0.925 ± 0.062 ^c^	3.123 ± 0.088 ^c^

Means with the same letter are not significantly different (*p* < 0.05).

**Table 2:** Main kinetic parameters, characterizing the TGSO autoxidation process at 80 °C in the presence of ferulates.

Feulates	Antioxidant Efficacy		Antioxidant Reactivity		Antioxidant Capacity	
	IP_A_ (h)	PF (-)	R_A_ ∙ 10^−6^ (mol/s)	ID (-)	R_m_∙ 10^−8^ (mol/s)	RR_m_∙ 10^−2^ (-)
Ferulic acid	5.0 ± 0.5 ^a^	3.9	1.3 ± 0.1 ^b^	4.6	5.6	4.3
*iso*-Ferulic acid	1.7 ± 0.1 ^b^	1.3	2.7 ± 0.3 ^a^	3.2	16.3	6.0
Coniferyl aldehyde	5.3 ± 0.5 ^a^	4.1	1.2 ± 01 ^b^	5.0	5.2	4.3
Methyl ferulate	5.7 ± 0.5 ^a^	4.4	1.3 ± 0.1 ^b^	4.6	4.9	3.5
Ethyl ferulate	5.7 ± 0.5 ^a^	4.4	1.3 ± 0.1 ^b^	4.6	4.9	3.5

IP_A_—induction period in presence of antioxidant; PF—protection factor; R_A_—initial rates of lipid autoxidation in presence of antioxidant; ID—inhibition degree; R_m_—main rate of antioxidant consumption; RR_m_—relative main rate of antioxidant consumption. Means with the same letter are not significantly different (*p* < 0.05).
